# Regulation of Fear Extinction in the Basolateral Amygdala by Dopamine D2 Receptors Accompanied by Altered GluR1, GluR1-Ser845 and NR2B Levels

**DOI:** 10.3389/fnbeh.2017.00116

**Published:** 2017-06-20

**Authors:** Yan-Wei Shi, Bu-Fang Fan, Li Xue, Jia-Ling Wen, Hu Zhao

**Affiliations:** ^1^Faculty of Forensic Medicine, Zhongshan School of Medicine, Sun Yat-sen UniversityGuangzhou, China; ^2^Guangdong Province Key Laboratory of Brain Function and Disease, Zhongshan School of Medicine, Sun Yat-sen UniversityGuangzhou, China; ^3^Guangdong Province Translational Forensic Medicine Engineering Technology Research Center, Zhongshan School of Medicine, Sun Yat-sen UniversityGuangzhou, China

**Keywords:** fear extinction, basolateral amygdala, D2 receptor, GluR1, GluR1-Ser845, NR2B

## Abstract

The amygdala, a critical structure for both Pavlovian fear conditioning and fear extinction, receives sparse but comprehensive dopamine innervation and contains dopamine D1 and D2 receptors. Fear extinction, which involves learning to suppress the expression of a previously learned fear, appears to require the dopaminergic system. The specific roles of D2 receptors in mediating associative learning underlying fear extinction require further study. Intra-basolateral amygdala (BLA) infusions of a D2 receptor agonist, quinpirole, and a D2 receptor antagonist, sulpiride, prior to fear extinction and extinction retention were tested 24 h after fear extinction training for long-term memory (LTM). LTM was facilitated by quinpirole and attenuated by sulpiride. In addition, A-amino-3-hydroxy-5-methyl-4-isoxazolepropionic acid receptor glutamate receptor 1 (GluR1) subunit, GluR1 phospho-Ser845, and *N*-methyl-D-aspartic acid receptor NR2B subunit levels in the BLA were generally increased by quinpirole and down-regulated by sulpiride. The present study suggests that activation of D2 receptors facilitates fear extinction and that blockade of D2 receptors impairs fear extinction, accompanied by changes in GluR1, GluR1-Ser845 and NR2B levels in the amygdala.

## Introduction

Fear extinction refers to the decrease in conditioned fear responses that occurs with repeated presentation of the unreinforced conditioned fear stimulus (CS) (Milad and Quirk, 2012). It has been reported that extinction represents new learning and that its expression can be easily disrupted. Over the past decades, research focusing on the behavioral and psychological aspects of extinction have indicated that extinguished fear returns spontaneously after the passage of time (Baum, 1988) or is “reinstated” by the presentation of the unconditioned fear stimulus (US) alone (Rescorla and Heth, 1975) or is “renewed” when the CS is presented in a context that is distinct from the context of the extinction (Bouton and King, 1983). Thus, identifying the mechanism of extinction and determining the best ways to facilitate extinction are vital for efficient behavioral therapy.

The amygdala is a critical structure for extinction and comprises the basolateral complex (BLA) and the central nucleus (CeA). Specifically, the BLA is a region where the CS and the US converge, enabling the CS to elicit freezing and other related conditional responses (Sotres-Bayon et al., 2006). Recent behavioral and pharmacological studies have reported participation of the BLA in fear extinction learning, including fear responses caused by the infusion of *N*-methyl-D-aspartic acid (NMDA) receptor (NMDAR) antagonists, mitogen-activated protein kinase (MAPk), or bupivacaine anesthetic (Herry et al., 2006; Kim et al., 2007; Sotres-Bayon et al., 2007). Additionally, consolidation involves activation of the phosphoinositide-3 kinase pathway in the BLA, synthesis of new proteins, and the expression of immediate early genes (Lin et al., 2003; Herry and Mons, 2004). These results have demonstrated that the BLA is involved in the acquisition and consolidation of extinction.

Dopamine (DA) is a transmitter that potently modulates the mechanisms underlying states of fear and anxiety (Millan, 2003). DA receptors are divided into two major subclasses: D1-like (D1 and D5) and D2-like (D2–D4) receptors (Vallone et al., 2000). Early reports have demonstrated that the systemic injection of SCH23390, a D1 receptor antagonist, inhibits fear-potentiated startle (FPS) (Davis et al., 1993). Conversely, studies of D1-deficient mice have suggested that D1 receptor fails to impair fear memories and that it facilitates fear extinction (El-Ghundi et al., 2001; Hikind and Maroun, 2008). However, compared with D1 receptor, few studies have examined the role of D2 receptor in extinction. Systematic D2 receptors have been reported to facilitate the extinction of conditioned fear in rats (Ponnusamy et al., 2005). Regarding specific brain structures, infralimbic D2 receptors impair extinction (Mueller et al., 2010; Zbukvic et al., 2017). However, the detailed mechanism of fear extinction regulated by D2 receptors in the amygdala remains unclear.

The amygdala receives rich dopaminergic input from ventral tegmental area (VTA) neurons in the mesencephalon (Fallon and Ciofi, 1992). DA metabolism in the amygdala has been previously shown to increase in animals that have received a footshock or conditioned fear-arousing stimulus (Coco et al., 1992; Inoue et al., 1994). Based on the moderate density of D2 receptors in the amygdala (Boyson et al., 1986) and the regulation of amygdala or BLA D2 receptors in the formation and retention of newly acquired fear associations (Greba et al., 2001; de Oliveira et al., 2011), we hypothesized that the extinction of conditioned fear might be regulated by D2 receptors in the amygdala.

Numerous studies have demonstrated that DA modulates responses evoked by activation of glutamate receptors, including A-amino-3-hydroxy-5-methyl-4-isoxazolepropionic acid (AMPA) receptors and NMDA receptors. One basic mechanism that is thought to be involved in memory formation is synaptic plasticity mediated by AMPA receptors containing the glutamate receptor 1 (GluR1) subunit. Mice lacking the GluR1 subunit fail to express long-term potentiation (LTP) in the basal amygdala (Humeau et al., 2007). GluR1 in the BLA is thought to be involved in the plastic synaptic events that underlie fear extinction. However, the relationship between GluR1 and D2 in the BLA remains controversial. In the striatum, glutamatergic transmission has been shown to be potentiated in dopamine D2 receptor-knockout mice (Cepeda et al., 2001), and D2 receptor antagonists have been demonstrated to enhance GluR1 phosphorylation at Ser845 (Håkansson et al., 2006). In striatal medium-sized spiny neurons (MSNs), the D2 receptor antagonist quinpirole has been reported to reduce AMPA current amplitudes (Hernández-Echeagaray et al., 2004). However, whether amygdala D2 receptor activation can modulate postsynaptic AMPA responses or receptor phosphorylation, even in fear extinction, remains unclear.

*N*-methyl-D-aspartic acid receptors are also crucial for many forms of learning and synaptic plasticity (Martin et al., 2000). Electrophysiological and behavioral pharmacological studies have established that NMDARs in the BLA play important roles in synaptic plasticity and fear conditioning (Blair, 2001). Systemic blockade of NMDARs impairs fear extinction (Santini et al., 2001), while systemic augmentation of these receptors facilitates fear extinction (Walker et al., 2002). Intra-BLA blockade of NMDARs disrupts LTP and interferes with the acquisition of auditory fear memory (Fendt, 2001; Bauer et al., 2002). NMDAR protein levels and currents are also down-regulated in the amygdala during the maintenance of fear memory (Zinebi et al., 2003). However, the distinct functional roles of NMDARs depend on their subunit composition (Müller et al., 2009). Among the several NR2 subtypes, NR2A and NR2B receptors are found in the amygdala (Monyer et al., 1992; Lopez de Armentia and Sah, 2003). NR2B is required for acquisition of auditory fear memory. Additionally, intra-BLA ifenprodil, a non-competitive, selective antagonist of NR2B NMDARs (Williams, 2001), impairs the acquisition of fear extinction. Quinpirole treatment inhibits NMDAR signaling in both the hippocampus and the PFC (Beazely et al., 2006; Gao and Wolf, 2008). However, the relationship between D2 and NR2B in the amygdala is still unclear.

The present study aimed to examine the effects of bilateral intra-BLA infusion of a D2 receptor agonist, quinpirole, and a D2 receptor antagonist, sulpiride, before extinction training on the long-term memory (LTM) of fear extinction by using the freezing level of rats as an index of fear. The AMPAR GluR1 subunit, GluR1 phospho-Ser845, and the NMDAR NR2B subunit were evaluated in the BLA via Western blotting.

## Materials and Methods

### Subjects

A total of 174 adult male Sprague-Dawley rats (220–250 g), which were included in the final analysis, were obtained from the Zhongshan School of Medicine, Sun Yat-Sen University, and housed under a 12/12-h light/dark cycle (lights on at 6:00 am) in Plexiglass cages under controlled temperature and humidity. Food and water were provided throughout the duration of the experiments. All procedures were approved by the Institutional Animal Care and Use Committee of the Zhongshan School of Medicine, Sun Yat-Sen University, in accordance with the National Institutes of Health Guide for the Care and Use of Laboratory Animals.

### Behavioral Procedures

#### Apparatus

The rats underwent acclimation, fear conditioning, extinction, and testing in two different chambers. Acclimation and fear conditioning occurred in chamber A, which was constructed of aluminum and Plexiglass walls (30 cm × 24 cm × 21 cm; Coulbourn Instruments, Lehigh Valley, PA, United States). The chamber was lit with a single house light and enclosed within a sound-isolation cubicle. The floor of each chamber consisted of 19 stainless steel rods (4 mm in diameter) that were spaced 1.5 cm apart (center to center). Foot shocks were used as US, induced by foot rods wired to a shock source. The acoustic CS was delivered by a speaker on one wall of the chamber. Both the conditioning box and floor were cleaned with 70% ethanol before and after each session. Extinction and testing occurred in chamber B. Three walls and the floor were covered with white paper and cleaned with 1% acetic acid before and after each session. To maximize discrimination between the two contexts, the light color was changed from white to red. Two different contexts were used for conditioning and extinction to condition the rats specifically to the tone and to minimize the effect of context (Hikind and Maroun, 2008). Above each chamber, closed-circuit video cameras recorded the behavior of each rat for behavioral scoring.

#### Fear Conditioning Procedure

On Day 1, all rats were first exposed to five habituation trials (CS-alone presentation), followed by three conditioning trials (CS-US pairing) in chamber A on Day 2. The CS was a 30 s, 75 dB, 4 kHz tone that co-terminated with a 1 s, 1.0 mA footshock US for fear conditioning. The mean inter-trial interval (ITI) was 3 min (2–4 min range) throughout habituation and fear conditioning. Sixty seconds after conditioning, the rats were returned to their home cages and to the colony room.

#### Extinction Procedure

Twenty-four hours after the conditioning session, extinction training, including 40 CS-alone presentations, which were used to optimize the efficacy of extinction, was performed in chamber B. During this period, rats assigned to the experimental or control group were presented with 40 tones (30 s, 75 dB, 4 kHz; average 1.5 min ITI) without a footshock. Rats that showed ≤50% freezing during the first five tones were excluded from the subsequent study phases (Yang et al., 2006). The LTM of fear responses conditioned to the CS tone were tested 24 h after extinction training in chamber B. During the test period, the rats received five test tones (30 s, 4 kHz, 75 dB; average 3 min ITI) without a footshock. Freezing was continuously recorded during the extinction training and test sessions.

#### Data Collection and Analysis

Freezing was used to measure conditioned fear. It was continuously recorded during the conditioning session and was later scored to determine the degree to which the rats acquired the conditioned association. Behavioral data were recorded with digital video cameras, and freezing was quantified from digitized video images using FreezeView2 software.

Data were analyzed using one-way analysis of variance (ANOVA). *Post hoc* comparisons of means were performed using Turkey’s test for multiple comparisons. The level of statistical significance was set at *P* < 0.05. For analyses of within-session extinction, the data were collapsed into 10 blocks of 4 CS presentations per block (extinction blocks). The data are presented as the mean ± standard deviation (SD).

### Drugs

To identify the role of D2 in modulating fear extinction, a D2 agonist, quinpirole (0.25–1.0 μg/μl), and a D2 antagonist, sulpiride (0.5–2.0 μg/μl) (Sigma–Aldrich Co.), were dissolved in sterile physiological saline (0.9%) 30 min before the experiment. Each drug or saline was injected into the BLA 30 min prior to extinction training at a constant volume of 0.3 μl/site.

### Surgery and Intra-BLA Injection

The rats were anesthetized with sodium pentobarbital (50–60 mg/kg, i.p.) and placed in a stereotaxic frame. Cannulae were implanted into the BLA (2.8 mm posterior, 5.0 mm lateral, and 7.8 mm ventral to the bregma) (Paxinos and Watson, 2004). The cannulae were secured to anchor jeweler’s screws with dental acrylic. Infusion cannulae were replaced with dummy cannulae that were cut to extend 0.5 mm beyond the guide cannulae to prevent clogging. At the end of surgery, the animals were placed under a heat lamp to maintain their body temperature and were continuously observed until locomotion returned, at which time they were returned to their home cage.

After 7 days of recovery from surgery, the rats were subjected to habituation and fear conditioning and to extinction training on the following day (see above). Thirty minutes before extinction training, the rats received bilateral intra-BLA infusions of either saline or drug. Solutions were infused in freely moving rats at a rate of 0.25 μl/min through the infusion cannulae attached to a 1.0-μl Hamilton syringe via polyethylene tubing. The cannulae were left in place for an additional 3 min after infusion to allow the solution to diffuse away from the cannulae tip, after which the dummy cannulae were replaced. The rats were returned to their home cage, which was returned to the colony room.

### Locomotor Activity Test

An automated activity monitoring system from HBZH (China) was used to assess locomotor activity. Each rat was individually subjected to a locomotor activity test in a sensor-equipped chamber (100 cm × 100 cm × 50 cm) and allowed to explore for 1 h. One day later, all animals were returned to the open field, drug free, for 1 h. Locomotor activity was quantified by dividing the floor into four squares and scoring the line crossings during the first 10 min on each day. The sensors registered the activity of the animal using video-tracking technology. A video camera was placed on the metal grid cover of a home cage. Activity data were collected by a computer using HBZH’s specialized software.

### Protein Preparation and Quantification

Animals (*n* = 3 in each group) were anesthetized with sodium pentobarbital and decapitated immediately after testing. Then, coronal brain slices (400 μm thick) containing the amygdala were prepared. Tissue blocks from the BLA (∼22.5 mm from the bregma) were obtained from three consecutive 400-μm sections with the aid of a microscope, and approximately 80% of the identifying regions were included to reduce contamination by other tissues. The BLA samples were dissected as quickly as possible from the coronal slices, placed on ice under a dissecting microscope, and preserved in liquid nitrogen to avoid dephosphorylation and protein degradation. Samples were ground with a high-flux tissue grinder for 90 s. Then, the total protein concentrations in the supernatants were determined using a BCA Protein Assay Kit.

Western blotting was performed using WES, an automated capillary-based size-sorting system (ProteinSimple, San Jose CA, United States). This system first calculates the protein concentration and then displays a band image according to the calculated protein concentration, which is more accurate compared with manual work. All procedures were performed using the manufacturer’s reagents according to the user manual. Briefly, 8 μl diluted protein lysate was mixed with 2 μl of 5× fluorescent master mix and heated at 95°C for 5 min. The samples (1 μg), blocking reagent, wash buffer, primary antibodies, secondary antibodies, and chemiluminescent substrate were dispensed into the designated wells in a manufacturer-provided microplate. The plate was loaded into the instrument, and protein was drawn into individual capillaries on a 25-capillary cassette provided by the manufacturer. Protein separation and immunodetection were automatically performed on the individual capillaries using the default settings. The data were analyzed using Compass software (ProteinSimple, San Jose, CA, United States). The primary antibodies were GluR1 (Millipore) and NR2B (Millipore), and GAPDH was used as a loading control (rabbit).

### Histology

**FIGURE 1:**
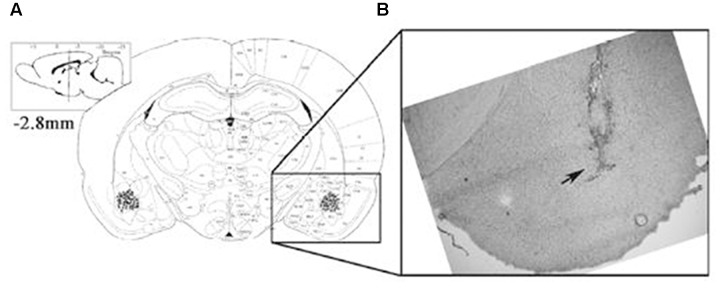
The location of the amygdala basolateral amygdala (BLA). Placement of the injection cannulae at the end of each experiment in rats administered intra-BLA injections of each drug and saline. **(A)** The injection sites are indicated by black dots. **(B)** The diagram shows a coronal view of the rat brain at 2.8 mm posterior to the bregma.

Following the retrieval test, all animals, except for those used for protein quantification, were administered an overdose of sodium pentobarbital and microinjected with methylene blue (1%, 1 μl) to mark the drug infusion site. The brain was then removed, and sections were examined to determine the location of the cannulae aimed toward the BLA area. The cannula locations were verified using a rat brain atlas. Only rats with cannula tips at or within the boundaries of the BLA were included in data analyses (**Figure 1**).

## Results

### Determining Optimal Dosing for Sulpiride and Quinpirole

**FIGURE 2:**
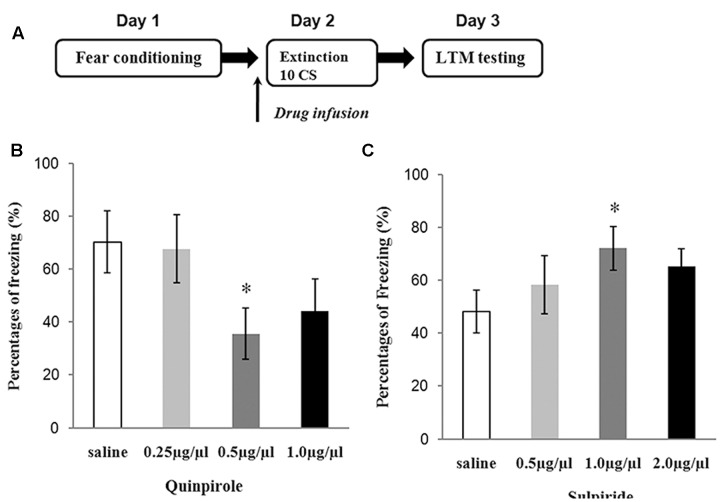
Experiments to determine the optimal doses of sulpiride and quinpirole. **(A)** Protocol for dose-determining experiments. **(B)** Average freezing during five CS presentations in the test for quinpirole (^∗^*P* < 0.05) (*n* = 6 or 7/group). **(C)** Average freezing during five CS presentations in the test for sulpiride (^∗^*P* < 0.05) (*n* = 6 or 7/group).

Three doses of sulpiride and 83 rats were used to test the effects of drugs on fear extinction. Saline and different doses of sulpiride were injected 30 min before 10 CS presentations. One day later, the freezing behavior of the animals was tested in the extinction context (**Figure 2A**). The ANOVA results were significant [*F*_(3,38)_ = 3.12, *P* < 0.05], and sulpiride significantly blocked extinction at the 1.0 μg/μl dose (*P* < 0.05, compared with the saline group) and was thus used in subsequent analyses (**Figure 2C**). The experimental protocol for quinpirole was the same as that for sulpiride. The ANOVA results were also significant [*F*_(3,37)_ = 4.01, *P* < 0.05], and 0.5 μg/μl was chosen for use in subsequent analyses because it was found to effectively facilitate fear extinction (*P* < 0.05, compared with the saline group) (**Figure 2B**).

### Spontaneous Locomotor Activity

**FIGURE 3:**
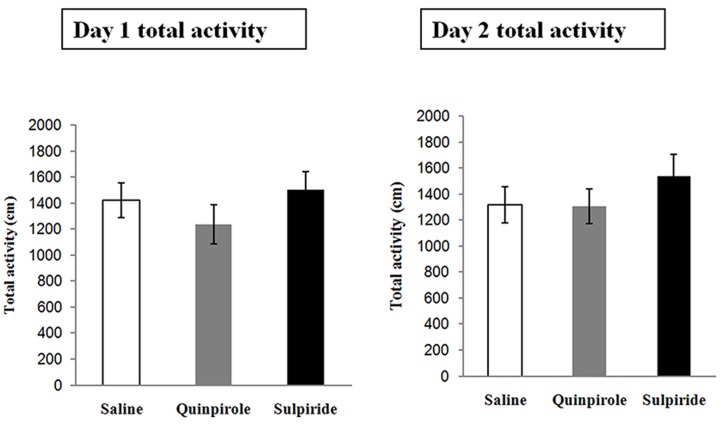
Effects of the optimal doses of sulpiride and quinpirole on locomotion. Rats were injected with saline (*n* = 6), quinpirole (*n* = 6) or sulpiride (*n* = 6) before being placed in the open field for 1 h on Day 1. On Day 2, they were returned, drug free, to the open field.

This experiment was designed to examine whether the selected doses of quinpirole and sulpiride affected spontaneous activity to minimize the chemical effects on the locomotion of individual rats. A total of 18 rats were injected with saline (*n* = 6), sulpiride (*n* = 6), or quinpirole (*n* = 6) and placed in an open field for 1 h on Day 1 [*F*_(2,15)_ = 1.24, *P* = 0.31]. On Day 2, the rats were returned to the open field without being administered a drug. Locomotor activity did not differ among the rats administered saline, sulpiride, or quinpirole in the drug-free test on Day 2 [*F*_(2,15)_ = 0.96, *P* = 0.57], indicating that there were no residual effects on motor activity that might account for the differences in freezing that we observed after extinction training in the above experiments (**Figure 3**).

### The Role of D2 Receptors in the BLA on Fear Extinction

**FIGURE 4:**
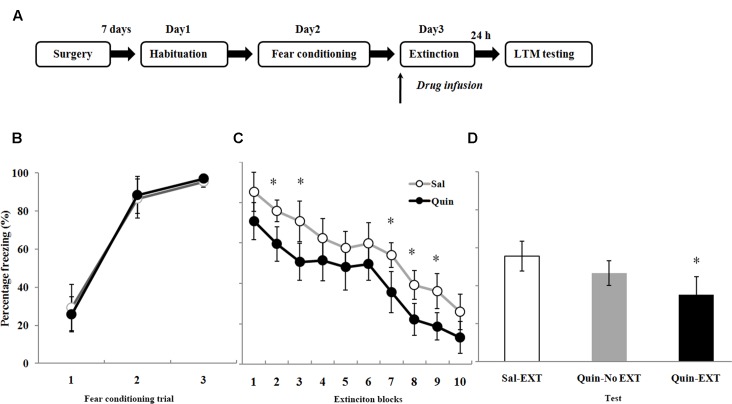
The effects of quinpirole microinfusion to the BLA before extinction training on long-term memory (LTM). **(A)** The protocol for drug administration. **(B)** Rats were presented with three pairings of tone and footshock in the conditioning chamber. **(C)** Administration of quinpirole before extinction training facilitated within-session extinction (^∗^*P* < 0.05). **(D)** The freezing levels of LTM after fear extinction training following quinpirole administration. The LTM level of the Quin-EXT group (*n* = 15) was also significantly lower than that of the Sal-EXT group I (*n* = 13, ^∗^*P* < 0.05).

A total of 70 rats underwent fear conditioning acclimation and fear conditioning on Days 1 and 2, respectively, and were divided into six groups according to which drug was microinfused into the BLA before extinction training on Day 3 (**Figure 4A**): the D2 receptor agonist quinpirole (Quin-EXT group, *n* = 15) vs. the saline group (Sal-EXT group I, *n* = 13) and the D2 receptor antagonist sulpiride (Sul-EXT group, *n* = 15) vs. the saline group (Sal-EXT group II, *n* = 14). In addition, groups that received quinpirole (Quin-No EXT group, *n* = 6) or sulpiride (Sul-No EXT group, *n* = 7) in the absence of extinction were also used to determine whether the effects on fear extinction were due to the drugs themselves or to the effects of the drugs combined with extinction.

**FIGURE 5:**
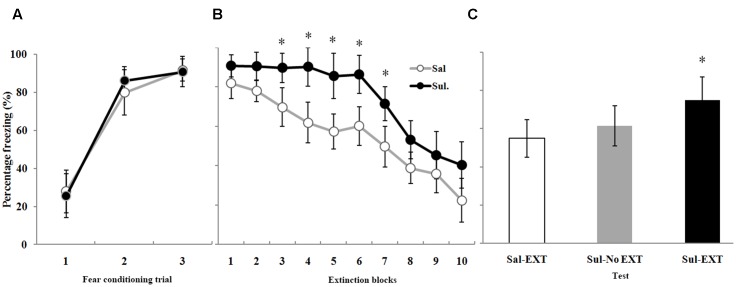
The effects of sulpiride microinfusion to the BLA before extinction training on LTM. **(A)** Rats were presented with three pairings of tone and footshock in the conditioning chamber. **(B)** Administration of sulpiride before extinction training impaired within-session extinction (^∗^*P* < 0.05). **(C)** The total freezing levels of LTM after fear extinction training following sulpiride administration. The LTM level of the Sul-EXT group (*n* = 15) was also significantly higher than that of the Sal-EXT group II (*n* = 14, ^∗^*P* < 0.05).

All rats increased their freezing levels during the conditioning process and did not differ between the group (**Figures 4B**, **5A**). Fear responses conditioned to the CS tone were tested at 24 h after extinction training (LTM). ANOVA revealed that both quinpirole [*F*_(2,31)_ = 6.36, *P* < 0.05] and sulpiride [*F*_(2,33)_ = 6.38, *P* < 0.05] had significant effects on freezing levels. A *post hoc* comparison confirmed that the freezing level of the Sal-EXT group (mean ± SD: 55.6 ± 7.9%) was significantly higher than that of the Quin-EXT group (mean ± SD, 35.4 ± 9.5%, *P* = 0.006) but that the Quin-No EXT group (mean ± SD, 46.7 ± 6.4%) exhibited no significant differences in the freezing level compared with the Quin-EXT group (*P* = 0.20) and Sal-EXT group (*P* = 0.16) in the extinction test (**Figure 4D**). ANOVA also revealed a significant effect of extinction block (*P* < 0.05), and an effect of drug at extinction blocks 2, 3, and 7–9 was observed, with the quinpirole group exhibiting less freezing than the saline group (all *P* < 0.05) (**Figure 4C**).

As expected, sulpiride induced the opposite effect. Fear expression was clearly decreased in the Sal-EXT group (mean ± SD, 54.8 ± 9.8%) compared with the Sul-EXT group (mean ± SD, 74.7 ± 12.2%) (*P* = 0.008). However, fear expression did not significantly differ between the Sul-No EXT group (mean ± SD, 61.4 ± 10.6%) and the Sul-EXT group (*P* = 0.057) and Sal-EXT group II (*P* = 0.55) (**Figure 5C**). ANOVA showed a significant effect of extinction block (*P* < 0.05), and an effect of drug at extinction blocks 3–7 was also observed, with the sulpiride group exhibiting more freezing than the saline group (all *P* < 0.05) (**Figure 5B**). Taken together, these results suggested that quinpirole facilitated fear extinction and that blockade of D2 receptors impaired fear extinction.

### GluR1 and GluR1-Ser845 Protein Levels in the BLA after Extinction Retrieval

**FIGURE 6:**
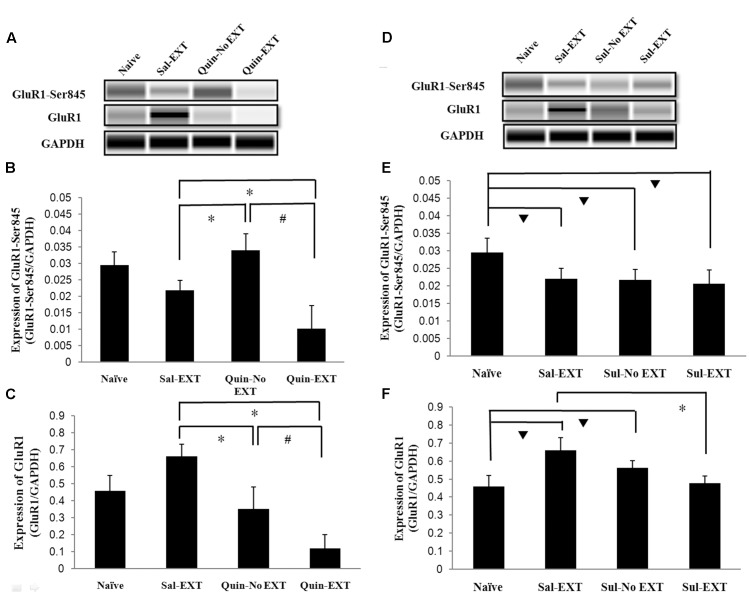
The effects of quinpirole and sulpiride infusion into the BLA before extinction training on GluR1 and GluR1-Ser845 levels after the LTM test. **(A–C)** Quinpirole rats showed decreased GluR1-Ser845 and GluR1 levels. ^∗^*P* < 0.05 vs. the Sal-EXT group. ^#^*P* < 0.05 vs. the Quin-No EXT group. **(D–F)** Sulpiride rats showed decreased GluR1 protein expression but no significant difference in GluR1-Ser845 levels. ^∗^*P* < 0.05 vs. the Sal-EXT group. 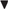
*P* < 0.05 vs. the naïve group. *n* = 3 in each group.

In the protein quantification experiment, a naïve group, which did not receive any drug treatment or behavior training, was established to evaluate baseline protein levels. GluR1 and GluR1-Ser845 protein expression levels in the BLA after 24 h of extinction retrieval were examined to confirm the relationship between D2 receptors and GluR1 in fear extinction. Western blotting showed that the facilitation of extinction with quinpirole was accompanied by reduced GluR1 protein levels in the BLA [*F*_(3,8)_ = 16.66, *P* = 0.001]. *Post hoc* tests showed that quinpirole significantly down-regulated GluR1 expression in the Quin-EXT group compared with the Sal-EXT group (*P* < 0.05). Furthermore, GluR1 expression was higher in the Quin-No EXT group than in the Quin-EXT group (*P* = 0.018) but was lower than in the Sal-EXT group (*P* = 0.004), revealing a combined effect of quinpirole and extinction on GluR1 expression (**Figures 6A,C**). GluR1-Ser845 levels [*F*_(3,8)_ = 13.12, *P* = 0.002] were much higher in the Quin-No EXT group than in the Sal-EXT (*P* = 0.018) and Quin-EXT groups (*P* < 0.05). However, GluR1-Ser845 levels were also lower in the Quin-EXT group than in the Sal-EXT group (*P* = 0.02) (**Figures 6A,B**).

For sulpiride, although GluR1 expression levels in the Sal-EXT, Sul-No EXT, and Sul-EXT groups showed similar trends as that in the quinpirole group (i.e., Sal-EXT group > Sul-No EXT group > Sul-EXT group), the Sul-No EXT group exhibited higher GluR1 levels than the naïve group (Sul-No EXT vs. naïve, *P* = 0.046), in contrast with the Quin-No EXT and Quin-EXT groups. However, GluR1 expression in the Sal-EXT group was still much higher than that in the Sul-EXT group (*P* = 0.003) (**Figures 6D,F**). In contrast with GluR1 expression, GluR1-Ser845 expression levels in the Sal-EXT, Sul-No EXT, and Sul-EXT groups were all significantly lower than that in the naïve group (all *P* < 0.05); however, the differences were not significant among all groups (all *P* > 0.05) (**Figures 6D,E**).

### NR2B Protein Levels in the BLA after Extinction Retrieval

**FIGURE 7:**
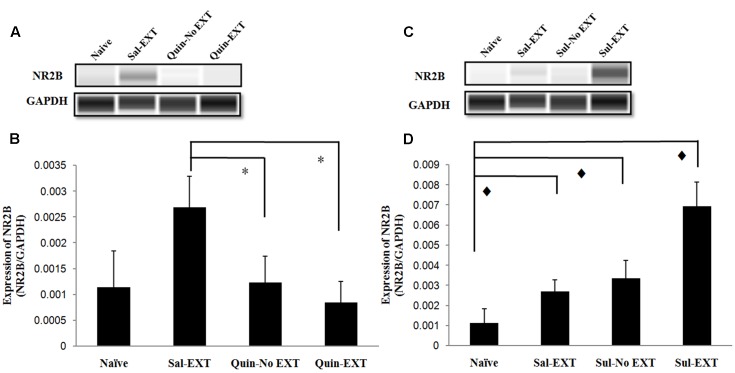
The effect of infusion of quinpirole or sulpiride into the BLA before extinction training on NR2B expression levels after the LTM test. **(A,B)** Quinpirole rats showed decreased NR2B expression. ^∗^*P* < 0.05 vs. the Sal-EXT group. **(C,D)** Sulpiride rats showed increased NR2B expression. 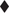
*P* < 0.05 vs. the Sul-EXT group. *n* = 3 in each group.

Because NR2B in the BLA plays an important role in fear extinction, we also examined NR2B expression after the LTM test. In the quinpirole experiment [*F*_(3,8)_ = 6.47, *P* = 0.016], NR2B expression exhibited a similar decrease as GluR1 expression, i.e., Sal-EXT group > Quin-No EXT group > Quin-EXT group (*post hoc* comparison: Sal-EXT vs. Quin-No EXT, *P* = 0.013; Sal-EXT vs. Quin- EXT, *P* = 0.04; Quin-No EXT vs. Quin- EXT, *P* = 0.423) (**Figures 7A,B**).

Moreover, in the sulpiride treatment experiment [*F*_(3,8)_ = 23.89, *P* = 0.000], the Sul-EXT group exhibited much higher NR2B expression than the other three groups (*post hoc* comparison: Sul-EXT vs. Sul-No EXT, *P* = 0.001; Sul-EXT vs. Sal-EXT, *P* = 0.000; Sul-EXT vs. naive, *P* = 0.000), suggesting that the regulation of fear extinction by sulpiride is more dependent on NR2B than on GluR1 (**Figures 7C,D**).

## Discussion

The results of the present experiments demonstrated that intra-BLA infusion of quinpirole and sulpiride, a D2 receptor agonist and antagonist, respectively, before extinction training of rats, facilitated and attenuated freezing in response to CS presentation in LTM tests. Further investigation revealed that GluR1, GluR1-Ser845 and NR2B protein levels may underlie the mechanism by which D2 receptors regulate fear extinction.

The ability of quinpirole to facilitate fear extinction generally corresponds to previous findings of a decreased fear response. For example, systemic or intra-BLA injection of quinpirole has been reported to reduce the expression of conditioned fear and FPS (de Oliveira et al., 2006, 2011). However, Ponnusamy et al. (2005) and Zbukvic et al. (2017) have observed impaired extinction (i.e., an increased fear response in extinction retention) in mice after systemic administration of quinpirole. This disparity may be explained by the fact that quinpirole is a dopamine D2-like receptor agonist with similar affinity for D2 and D3 receptors (Kebabian et al., 1997). Consistent with previous reports (Collins et al., 2007), quinpirole produced a U-shaped dose-response curve. The biphasic nature of this dose-response curve is thought to be related to the induction of freezing by the activation of D3 receptors at smaller doses of quinpirole and the inhibition of freezing by activation of D2 receptors at larger doses of quinpirole. Furthermore, quinpirole can pass through the blood-brain barrier after intraperitoneal injection. Therefore, the amount of the drug that is functioning in the brain cannot be determined. Low and high doses may have opposite effects. In addition, the effects are inconsistent due to the variety of behavioral and pharmacological manipulations that have been used (Abraham et al., 2014).

An explanation of the facilitation of fear extinction by intra-BLA injection of quinpirole is that the effect of quinpirole may be linked to DA release in the BLA (Bull et al., 1990). Biochemical analyses of dialysate from the amygdala have revealed that DA release can be induced by electric footshock (Muller et al., 2009). This increased DA level excites BLA pyramidal cells, and a decrease in dopamine activity in the BLA reduces the expression of conditioned fear (Brandão et al., 2015). Because quinpirole inhibits DA release, we speculated that the reduction in DA suppresses the activity of pyramidal cells in the BLA.

To test whether the effects on fear extinction are due to the drugs themselves or to the effects of the drugs combined with extinction, we evaluated a group that received intra-BLA injection of quinpirole while experiencing fear conditioning without extinction. In the process of extinction learning, the freezing levels of the treated groups (i.e., the Quin-EXT and Sul-EXT groups) were significantly different from that of their respective Sal-EXT group with extinction block, with all rats showing initially high levels of CS-elicited freezing that decreased as extinction proceeded, indicating that not only extinction but also the drugs worked in the acquisition of within-session extinction. However, no significant differences between the Quin-EXT and Quin-No EXT groups or between the Sul-EXT and Sul-No EXT groups were observed in the test. Thus, it seems that extinction did not work. The potential reasons for these findings are as follows: (1) the small size of the No-EXT groups in our study may have affected the results; and (2) the role of extinction in the impairment or facilitation of freezing may have been masked by the drugs.

The sulpiride-induced impairment of the acquisition of fear extinction observed in the present study generally corresponds to previous reports in the literature. For example, the D2 receptor antagonist raclopride has been shown to impair extinction following injection into the infralimbic medial prefrontal cortex (IL) (Mueller et al., 2010). Haloperidol has also been demonstrated to block extinction after systemic or local injection into the nucleus accumbens (Holtzman-Assif et al., 2010). However, the opposite effect on fear extinction has been reported in a relevant study in which fear extinction was accelerated in mice after systemic sulpiride injection (Ponnusamy et al., 2005). The discrepancies among these studies have been more thoroughly discussed by Mueller et al. (2010), but a contributing factor could be the varying receptor affinities among different D2 antagonists or the differing CS presentation protocols used in the experiments. Furthermore, effects of the drugs on motor performance can be excluded because the same doses used in the present study did not affect motor performance in the open field. In addition, although the injection sites were confirmed and the microinjection speed and volume were limited, we cannot exclude the possibility that the drugs may spread beyond the intended brain region (Li et al., 2015).

Recent studies have indicated that both fear conditioning-induced neuronal plasticity and LTP at amygdala synapses share common mechanisms of induction and expression (Dityatev and Bolshakov, 2005). Differing contribution of AMPARs to the extinction learning process have been reported. Although AMPAR antagonists in the amygdala have no effect on extinction acquisition (Kim et al., 2017), electrophysiological experiments have demonstrated that fear extinction learning corresponds to a loss of calcium-permeable AMPA receptors (GluR1-containing) in the BLA (Clem and Huganir, 2010). As previously reported, auditory fear conditioning is accompanied by enhanced synaptic plasticity at auditory input synapses in the BLA, and extinction reverses both the enhanced synaptic efficacy and the conditioning-induced enhancement of surface AMPAR expression (Lin et al., 2009). Additionally, when rodents exhibit renewal, there is increased GluR2-lacking AMPA signaling in the LA (Lee et al., 2013; Park et al., 2014). Considering these results, we speculate that lower AMPAR expression is accompanied by less freezing behavior, which is in line with our results demonstrating that the facilitated extinction caused by D2 receptors decreased the expression of AMPARs. A comparison of GluR1 expression among the three groups in our study revealed that the Quin-No EXT group exhibited higher expression than the Quin-EXT group but lower expression than the Sal-EXT group. These results provide evidence that AMPAR removal at excitatory synapses in the LA underlies extinction (Kim et al., 2007; Lin et al., 2009) and that quinpirole decreases AMPAR GluR1 synaptic expression. Consistent with the behavioral data reported in the present study, quinpirole and extinction training acted in conjunction to decrease AMPAR GluR1 synaptic expression.

Thus far, few studies have examined the relationship between D2 receptors and AMPARs in the amygdala compared with other brain regions. In the striatal medium, haloperidol or eticlopride, both D2 receptor antagonists, induces GluR1 Ser845 phosphorylation by PKA without altering total GluR1 levels (Håkansson et al., 2006). However, in the PFC, D2 receptor activation decreases AMPAR GluR1 surface and synaptic expression levels (Sun et al., 2005). *In vitro* electrophysiological studies of the BLA have revealed that both effects of dopamine (e.g., reduced inhibition of projection neurons and increased inhibition of interneurons) are mediated by D2 receptors (Bissière et al., 2003; Kröner et al., 2005). If quinpirole activated D2 receptors in the amygdala, dopamine release would be inhibited, potentially increasing inhibition of projection neurons and reducing LTP induction, thus leading to less freezing behavior. Alternatively, one consequence of D2 receptor activation in the BLA is the rapid, direct depression of the excitability of BLA pyramidal neurons that results from the activation of GABA transmission and the modulation of Na^+^ conductance, which occurs by a similar mechanism as in the PFC (Gulledge and Jaffe, 1998, 2001). Unlike the striatal medium, not only total GluR1 levels but also phosphorylation of the PKA site Ser845 of GluR1 were altered in the present study. The decreased GluR1 and GluR1-Ser845 levels by quinpirole can be explained as follows: on the one hand, as in the mPFC, quinpirole in the BLA may decrease AMPAR GluR1 surface and synaptic expression levels. On the other hand, because LTP induction requires activation of cAMP-PKA, reversal of the conditioning-induced enhancement of synaptic efficacy by extinction may reduce cAMP-PKA activity, thereby decreasing GluR1-Ser845 levels. This model suggests an additional mechanism by which D2 receptors may depress neuronal excitability and plasticity and reduce AMPARs rather than by simply inhibiting PKA in the BLA. Additionally, previous studies have shown that renewed expression is related to Ser831 phosphorylation of the GluR1 subunit in the LA (Lee et al., 2013). In addition to the regulation of GluR1 expression by D2 receptors these findings may also suggest different signaling mechanisms of extinction and renewal.

Unexpectedly, although GluR1 expression levels in the Sul-No EXT and Sul-EXT groups were higher than that in the naïve group, whereas the Quin-No EXT and Quin-EXT groups showed lower expression, GluR1 expression showed a similar trend as that in the quinpirole-treated rats in the Sul-No EXT and Sul-EXT groups compared with the rats in the Sal-EXT group (i.e., GluR1 expression: Sal-EXT > Sul-No EXT > Sul-EXT; Sal-EXT > Quin-No EXT > Quin-EXT). One possible reason for these findings may be the dosage used in our experiment. Because low concentrations of sulpiride significantly inhibit eEPSC transmission of DA, whereas high concentrations prevent this inhibition *in vitro* (Darvish-Ghane et al., 2016), sulpiride may exert bidirectional regulation over eEPSC transmission. Although the dosage chosen in our experiment was optimal for *in vivo* studies, it may not have been sufficient to increase eEPSC transmission, especially for GluR1 expression. Potential explanations for these differences may lie in the degree of spontaneous network synaptic inputs that impinge on the neurons *in vivo* or in brain slices.

As with AMPAR GluR1, BLA NR2B is also involved in LTP induction and the acquisition of auditory fear memory. Several lines of evidence have shown that NR2B in the amygdala is essential for fear conditioning. For example, intra-amygdala blockade of NR2B using ifenprodil or interference with NR2B-mediated signaling using a polyamine inhibitor or via NR2B phosphorylation through knock-in mutation impairs LTP, fear-memory formation and fear-memory extinction (Rodrigues et al., 2001; Zinebi et al., 2003; Nakazawa et al., 2006; Sotres-Bayon et al., 2007). These studies have revealed that NR2B is involved in synaptic strength in auditory fear memory (Zhang et al., 2008). Consistent with these results, NR2B expression in the present study was generally decreased by quinpirole treatment, accompanied by enhanced extinction with a lower freezing level. In contrast, NR2B expression was increased by sulpiride, accompanied by blocked extinction with a higher freezing level. As protein expression in the Sal-EXT group was lower than that in the Sul-EXT group, the inductive effect of sulpiride on NR2B expression counteracted the extinction-induced decrease in NR2B. Furthermore, as NMDARs are critically involved in different forms of synaptic plasticity, including LTP, LTD, and depotentiation, and because NR2B recruitment depends on the strength of conditioning (Zhang et al., 2008), further experiments are necessary to determine which form of plasticity is required for extinction learning under the regulation of NR2B.

The relationship between D2 and NMDA receptors in the amygdala has not been previously reported. However, in CA1 pyramidal neurons, activation of D2-class dopamine receptors by quinpirole has been shown to depress the excitatory transmission mediated by NMDA-type glutamate receptors (Kotecha et al., 2002). This depression results from the quinpirole-induced release of intracellular Ca^2+^ and enhanced Ca^2+^-dependent inactivation of NMDA receptors. In the neostriatum, D2Rs directly interact with NR2B and disrupt NR2B-CaMKII binding, thereby inhibiting Ser1303 phosphorylation (Liu et al., 2006). As previously reported, LTP induction in auditory pathways in auditory fear memory in the amygdala is dependent on Ca^2+^ influx into the postsynaptic cell (Dityatev and Bolshakov, 2005). Ca^2+^ ions, which are required for LTP induction, enter the postsynaptic cell through NMDA receptors. Whether D2Rs modulate NR2B through Ca^2+^ ions or by some other mechanism remains to be determined. In addition, based on the increased freezing level in the fear extinction LTM test with sulpiride and increased NR2B expression, we speculate that impairment of fear extinction by sulpiride may depend more on NR2B than on GluR1.

## Conclusion

The present results illustrated the importance of amygdala D2 receptors in fear extinction. The freezing levels decreased in response to quinpirole, a D2 agonist, in the later retrieval of extinction, whereas the freezing levels increased in response to sulpiride, a D2 antagonist. The effects of quinpirole and sulpiride were accompanied by changes in AMPAR GluR1 subunit, GluR1 phospho-Ser845, and NMDAR NR2B subunit levels.

## Author Contributions

HZ contributed to the conception of the work. Y-WS designed and collected the data; B-FF, LX, and J-LW collected the data; Y-WS and B-FF analyzed the data; and Y-WS wrote the article. All authors discussed the results and commented on the manuscript. All authors approved the final version of the manuscript.

## Conflict of Interest Statement

The authors declare that the research was conducted in the absence of any commercial or financial relationships that could be construed as a potential conflict of interest. The reviewer SL and handling Editor declared their shared affiliation, and the handling Editor states that the process nevertheless met the standards of a fair and objective review.
